# Concurrent superior semicircular canal dehiscence and endolymphatic hydrops: A novel case series

**DOI:** 10.1016/j.ijscr.2020.12.074

**Published:** 2020-12-26

**Authors:** Michael Johanis, Russell De Jong, Tyler Miao, Leslie Hwang, Meachelle Lum, Taranjit Kaur, Shelby Willis, John J. Arsenault, Courtney Duong, Isaac Yang, Quinton Gopen

**Affiliations:** a Department of Head and Neck Surgery, Ronald Reagan UCLA Medical Center, Los Angeles, CA, United States; b Department of Neurosurgery, Ronald Reagan UCLA Medical Center, Los Angeles, CA, United States; c Office of the Patient Experience, Ronald Reagan UCLA Medical Center, Los Angeles, CA, United States; d UCLA Jonsson Comprehensive Cancer Center, Ronald Reagan UCLA Medical Center, Los Angeles, CA, United States; e Los Angeles Biomedical Research Institute (LA BioMed), at Harbor-UCLA Medical Center, Torrance, California, USA; f Department of Surgery at Harbor-UCLA Medical Center, Torrance, CA, United States

**Keywords:** Superior semicircular canal dehiscence, Endolymphatic hydrops, Meniere’s disease, CT, MRI, Case series

## Abstract

**Introduction:**

Superior semicircular canal dehiscence (SSCD) is characterized by CT-confirmed bony erosion over the superior semicircular canal, creating vestibular and auditory symptoms. Endolymphatic hydrops (EH) is characterized by an MRI-confirmed excess of endolymph within the scala media that distorts the membranous labyrinth. While there is overlap in symptoms, the two diseases result from different pathophysiologies and require different interventions.

**Presentation of cases:**

A retrospective chart review was conducted at the University of California, Los Angeles on a database of 270 adult SSCD patients, gathered between March 2011 and February 2020. A review of clinical notes, post-operative findings, and imaging was performed for 16 patients who had both CT-confirmed SSCD and an MRI of the internal auditory canal (IAC). Three cases of concurrent SSCD and EH were identified. Medical and surgical history, symptom progression pre- and post-operatively, and treatment outcomes were gathered. One patient’s symptoms were resolved via mycophenolate mofetil, another’s via hydrochlorothiazide, and the third’s via hydrochlorothiazide and bilateral hearing aids.

**Discussion:**

Post-surgical persistence of SSCD symptoms that are mutually shared with EH is the strongest indicator that a physician should investigate for concurrent EH. VEMP and audiogram testing in these cases can be misleading and should not be relied on as rule-in or rule-out tests.

**Conclusion:**

Concurrent SSCD and EH is a rare but treatable entity. Physicians should consider ordering an MRI of the IAC if SSCD patients’ symptoms persist or recur after a successful surgery.

## Highlights

• SSCD and EH are two uncommon inner ear disorders.
• UCLA is a high-volume otologic referral center with the most SSCD cases worldwide.
• Three patients presented with both SSCD and EH, which have overlapping symptoms.
• Post-surgical persistence of shared symptoms may prompt an investigation for EH.
• Concurrent SSCD and EH is a rare but treatable entity.

## 1 Introduction

Superior semicircular canal dehiscence (SSCD) and endolymphatic hydrops (EH) are two uncommon inner ear disorders. SSCD is characterized by an absence of bone overlying the superior semicircular canal [1]. Sound normally enters and exits the inner ear through the oval window and round window, respectively [2]. SSCD patients’ third window in the labyrinth causes various auditory and vestibular pathology such as amplification of internal sounds, autophony, pulsatile tinnitus, aural fullness, hearing loss, tinnitus, sound- and pressure-induced vertigo, hyperacusis, oscillopsia, nausea, and headaches [[3], [4], [5], [6], [7], [8]]. Carey et al.’s analysis of 1,000 cadaveric temporal bones from 596 adults revealed that approximately 2.0% of adults have either an absent or extremely thin bone (< 0.1 mm) overlying the canal, although the incidence of symptomatic patients is significantly lower [9].

EH is characterized by an excessive buildup of endolymph within the scala media, also known as the cochlear duct [10]. Symptoms include episodic vertigo, hearing loss, tinnitus, hyperacusis, nausea, and headaches [11]. These symptoms result from membranous labyrinth distortion, which in turn causes distortion of the delicate neural structures responsible for balance and hearing. The characteristic acute symptoms stem from a pressure-induced rupture in the basilar, also known as Reissner’s, membrane [12]. This creates mixing of perilymph and endolymph, causing depolarization blockade and loss of function which manifests as vertigo [13]. In the United States, the incidence of EH is variable based on age, from 9 per 100,000 in the below-18 age group to 440 per 100,000 in the 65-and-older group [14].

While there is significant overlap in symptomatology such as episodic vertigo, tinnitus, and hearing loss, the two diseases are derived from distinct pathophysiologies and therefore require different interventions. To the authors' best knowledge, this is the second case report of concurrent SSCD and EH, but the first to include a comprehensive, chronological review of clinical notes, audiograms, imaging, and treatment outcomes. Therefore, it is imperative to present this unique case series of patients with image-proven SSCD and EH in order to increase physician awareness of the potential overlap, as well as to facilitate diagnostic and prognostic efficiency. The work reported herein is in line with the PROCESS criteria [15,16].

## 2 Presentation of cases

A single-institution retrospective chart review of electronic medical records was conducted on 270 adult patients who presented with SSCD at the University of California, Los Angeles between March 2011 and February 2020. Diagnoses of SSCD were confirmed via a high-resolution computed tomography (CT) scan of the temporal bones. Imaging history was reviewed to find patients who had also undergone magnetic resonance imaging (MRI) of the internal auditory canal (IAC), a critical diagnostic tool for EH. This resulted in sixteen patients with CT-confirmed SSCD and an MRI of the IAC. A comprehensive review of clinical notes, post-operative findings, and imaging was performed for these sixteen patients. This revealed three patients who had image-confirmed EH.

Table 1 summarizes the symptoms of the 13 adult patients with a CT-confirmed diagnosis of SSCD and negative MRI IAC scans for EH. MRIs were ordered for these patients based on their individual symptomatology. Similar to the three patients with both diseases, most patients suffered from cardinal symptoms of EH such as tinnitus, aural fullness, vertigo, and disequilibrium. Only four patients exhibited hearing loss, as opposed to all of the EH confirmed patients.

**Table 1:** Compendium of the 13 adult patients with SSCD, confirmed via a high-resolution CT scan of the temporal bones, who had negative MRI IAC scans for EH.

Patient number	Age	Sex	SSCD Laterality	Pulsatile Tinnitus	Tinnitus	Amplification of Internal Sounds	Autophony	Aural Fullness	Hyperacusis
1	54	M	L	+			+	+	
2	36	F	B	+				+	
3	57	F	B	+		+		+	+
4	51	F	B	+		+	+	+	
5	47	M	L	+					
6	42	F	B		+	+	+	+	+
7	72	M	L						+
8	64	F	B	+			+	+	
9	55	F	L					+	
10	62	F	B	+					
11	59	M	L						
12	56	F	B	+		+		+	
13	59	F	R		+	+			

Patient number	Age	Sex	SSCD Laterality	Tullio Phenomenon	Nystagmus	Headache	Brain Fog	Vertigo	Balance Disequilibrium	Hearing Loss
1	54	M	L		+			+	+	
2	36	F	B				+	+	+	
3	57	F	B					+	+	
4	51	F	B			+	+	+		
5	47	M	L				+	+		
6	42	F	B				+	+		+
7	72	M	L	+				+	+	+
8	64	F	B					+	+	+
9	55	F	L					+	+	
10	62	F	B					+	+	
11	59	M	L	+				+	+	
12	56	F	B			+		+		
13	59	F	R			+		+	+	+

A team of otolaryngologists and radiologists made all the diagnoses of SSCD and EH via the aforementioned scans, whereas medical students and undergraduates performed the retrospective chart review. SSCD surgeries at UCLA were performed by a neurosurgeon and otolaryngologist team via the middle cranial fossa (MCF) approach. All patients received maximal medical therapy before being considered for surgery.

### 2.1 Case 1

Patient 1 is a 50-year-old male who initially presented in September 2014, six months after a sudden onset of left-sided sensorineural hearing loss that was treated with antivirals and oral steroids. His tinnitus, hearing loss, lightheadedness, and fatigue persisted, stopping him from teaching. Prescribed hydrochlorothiazide dosage was doubled from 25 mg daily to 25 mg B.I.D., which improved his symptoms for two months until November, when they returned on the left side and the right side began to experience tinnitus and hearing loss. These symptoms were further accompanied by head pressure. The intensity of the pressure and the otologic symptoms grew in tandem. CT imaging of the temporal bones was ordered and confirmed a diagnosis of left SSCD, although vestibular evoked myogenic potentials (VEMP) testing was inconclusive. Prior to surgical correction in May 2015, MRI of the internal auditory canal and brain with contrast was ordered, revealing bilateral endolymphatic hydrops. Both cochlear ducts as well as the left saccule presented with dilation. On the other hand, neither his utricles nor right saccule appeared to be dilated. His lightheadedness subsided after SSCD repair, but his left-sided pulsatile tinnitus and hearing loss persisted. At the six-month postoperative check-up, he was still experiencing mild vertigo, balance disequilibrium, and dizziness. All of these symptoms were steroid-responsive. This constellation of continued symptoms prompted repeat IAC and brain MRIs as well as electronystagmography testing. This workup confirmed endolymphatic hydrops that were worse on the left side than the right. Inflammatory etiology was suspected and a referral to rheumatology was made, where the patient was prescribed mycophenolate mofetil (CellCept®️). This treatment greatly reduced his symptoms.

### 2.2 Case 2

Patient 2 is a 41-year-old female with a history of bilateral SSCD and eustachian tube dysfunction leading to double bilateral mastoidectomies. Her symptoms of vertigo, nausea, right ear deafness, and Tullio phenomenon began in January 2013, the morning after she felt a pop in her right ear while lifting a heavy object. She underwent a right-side round window plugging in May 2013 and left port placement in December 2013, both at an outside institution. The right window plug initially improved her pulsatile tinnitus and internal amplification of eye movement sounds, but both symptoms eventually recurred. She was prescribed acyclovir and prednisone for one week in April 2014. After no improvements, in the same month she was given 25 mg of hydrochlorothiazide daily. Once this dosage was increased to twice a day beginning in May 2014, her symptoms began to improve. After two months, however, her symptoms relapsed.

During her first visit to UCLA in January 2015, the patient reported bilateral pulsatile tinnitus, bilateral autophony, right ear hearing loss, left hyperacusis, amplification of internal sounds, chronic dizziness, and imbalance. CT imaging of the temporal bones demonstrated right SSCD, for which she underwent a middle fossa craniotomy in February 2015. At the two-month follow-up, she reported improvement of right autophony, right amplification of internal sounds, balance, and ear pain/pressure. There was no change in profound right deafness, but the patient complained of worsening left ear symptoms, namely amplification of internal sounds, hyperacusis, and autophony. This prompted a left SSCD repair via the MCF approach in May 2015. Weeks after her symptoms failed to resolve, an MRI of the internal auditory canal and brain with contrast revealed left endolymphatic hydrops, specifically due to a dilated left saccule. A follow-up in April 2016 revealed continued autophony but improvement of hyperacusis.

### 2.3 Case 3

Patient 3 is a 49-year-old male with bilateral SSCD and bilateral EH. He presented in February 2020 for evaluation of a plethora of otologic symptoms: bilateral pulsatile tinnitus, autophony, hyperacusis, hearing loss, tinnitus, amplification of internal sounds, aural fullness, dizziness, vertigo, balance disequilibrium, oscillopsia, head pressure, brain fog, headaches, fatigue, and migraines. His left utricle, saccule, and cochlear duct were unequivocally dilated, whereas his right saccule and right cochlear duct were likely dilated. He was given 12.5 mg hydrochlorothiazide daily for his vertigo and wears bilateral hearing aids for his hearing loss.

## 3 Discussion

SSCD is generally associated with conductive hearing loss below 2 kHz and bone conduction lines above 0 kHz [17]. EH commonly presents with sensorineural hearing loss, also below 2 kHz [18]. When overlaid, it is clear that the presentation of one disease may easily be masked or mimicked by the other. Table 2, Table 3, Table 4 summarize the audiogram data for the three patients with concurrent SSCD and EH. The audiograms of the four ears with both SSCD and EH are consistent with this idea, as hearing loss below 2 kHz is evident without any distinct characteristics suggestive of concurrent SSCD and EH. To complicate matters further, EH that affects the otolith organs can be a confounding variable to VEMP testing [19], one of the most useful tests in the diagnosis of SSCD.

**Table 2:** Patient 1’s pre-surgical audiogram data from September 23, 2014.

Patient 1		Frequency (Hz)					
		250	500	1000	2000	4000	8000
Bone Conduction (dB)	Left*	0	25	5	15	25	
	Right*, ‡	40	60	65	70	75	
Air Conduction (dB)	Left*	35	35	15	15	25	15
	Right*, ‡	105	110	110	110	110	

Discrimination Score	Left	100%					
	Right	N/A					
Speech Reception Threshold	Left	25 dB					
	Right	N/A					

* Indicates SSCD.

‡ Indicates EH.

**Table 3:** Patient 2’s post-surgical audiogram data from August 18, 2015.

Patient 2		Frequency (Hz)					
		250	500	1000	2000	4000	8000
Bone Conduction (dB)	Left*, ‡		5	5	30	20	
	Right*		5	5	10	10	
Air Conduction (dB)	Left*, ‡	25	15	15	30	50	30
	Right*		15	15	10	10	20

Discrimination Score	Left	88%					
	Right	100%					
Speech Reception Threshold	Left	15 dB					
	Right	5 dB					

* Indicates SSCD.

‡ Indicates EH.

**Table 4:** Patient 3’s pre-surgical audiogram data from September 25, 2019.

Patient 3		Frequency (Hz)					
		250	500	1000	2000	4000	8000
Bone Conduction (dB)	Left*, ‡		60	50	55	50	
	Right*, ‡		50	45	50	55	
Air Conduction (dB)	Left*, ‡	55	60	60	55	50	50
	Right*, ‡	55	55	55	50	55	60

Discrimination Score	Left	56%					
	Right	80%					
Speech Reception Threshold	Left	55 dB					
	Right	45 dB					

* Indicates SSCD.

‡ Indicates EH.

The symptoms of SSCD and EH overlap significantly, including vertigo, hearing loss, tinnitus, hyperacusis, nausea, and headache. These three cases demonstrate that patients with concurrent EH and SSCD represent a significant diagnostic challenge that requires a high level of clinical suspicion leading to specialized tests and imaging. As shown by our patients, there is no reliable single symptom or set of symptoms that can definitively diagnose concurrent SSCD and EH.

The most noteworthy characteristic of all three patients was the persistence or recurrence of symptoms after a successful surgical repair of SSCD. Despite each patient’s lengthy otologic history, their diagnoses were confirmed by MRI of the IAC. Post-surgical persistence of SSCD symptoms that are mutually shared with EH appears to be the strongest indicator for a physician to order an MRI of the IAC to assess the cochlear duct and otolith organs. VEMP and audiogram testing in these cases can be misleading and should not be relied on as rule-in or rule-out tests. Lastly, a recent retrospective study found that 9 of 33 (27%) SSCD ears also had EH [20]; thus, a future prospective study with a large sample size should determine the prevalence of concurrent SSCD and EH.

### 3.1 Limitations

This case series is limited by its small sample size, the rarity of the subject matter which may be magnified by patients who have gone undiagnosed, and its retrospective nature. Future projects will most likely be similarly limited, as this study was done at a high-volume otologic referral center with the largest cohort of SSCD patients to our knowledge. More research on concurrent SSCD and EH is encouraged due to the symptomatic overlap and management implications for these complex patients.

## 4 Conclusion

Our case series is the most in-depth compendium of concurrent SSCD and EH, increasing physician awareness of the coexistence of two uncommon inner ear diseases. Future work on concomitant SSCD and EH, perhaps designed to establish the prevalence of their coexistence, would contribute most to the literature via a prospective, randomized approach or via a large multicenter study to increase sample size and statistical power. Physicians should strongly consider ordering an MRI of the IAC if SSCD patients’ symptoms persist or recur after a successful surgery. Finally, the three cases demonstrate that concurrent SSCD and EH is an atypical diagnosis, but with appropriate intervention, can be treated.

## Declaration of Competing Interest

The authors report no declarations of interest.

## Funding

This research did not receive any specific grant from funding agencies in the public, commercial, or not-for-profit sectors.

## Ethical approval

This study was approved by the University of California, Los Angeles Institutional Review Board (IRB # 17-001563).

## Consent

Not applicable. In light of this IRB-approved case series being a retrospective chart review with no images of patients or other patient identifiers, this paper is exempt from written informed consent forms.

## Registration of research studies

researchregistry6384 available at: https://www.researchregistry.com/browse-the-registry#home/registrationdetails/5fe163fcaaea69001bb879d8/.

## Guarantor

Quinton Gopen, MD (Associate Professor at the UCLA Department of Head and Neck Surgery).

## Provenance and peer review

Not commissioned, externally peer-reviewed.

## CRediT authorship contribution statement

**Michael Johanis:** Conceptualization, Data curation, Formal analysis, Methodology, Writing - original draft, Writing - review & editing. **Russell De Jong:** Conceptualization, Data curation, Formal analysis, Methodology, Writing - original draft, Writing - review & editing. **Tyler Miao:** Data curation, Formal analysis, Methodology, Writing - original draft. **Leslie Hwang:** Data curation, Writing - original draft. **Meachelle Lum:** Formal analysis, Writing - review & editing. **Taranjit Kaur:** Writing - review & editing. **Shelby Willis:** Writing - review & editing. **John J. Arsenault:** Writing - review & editing. **Courtney Duong:** Writing - review & editing. **Isaac Yang:** Conceptualization, Formal analysis, Writing - review & editing. **Quinton Gopen:** Conceptualization, Formal analysis, Methodology, Writing - review & editing.
